# FDG PET/CT in Prostate Cancer: A Valuable Method to Detect the Primary and Metastatic Tumor Sites and to Monitor Cancer Response to Hormonal Therapy

**DOI:** 10.5812/numonthly.4539

**Published:** 2012-09-24

**Authors:** Alexander Hinev, Borislav Chaushev, Aneliya Klisarova

**Affiliations:** 1 Department of Surgery, Clinic of Urology, Varna Medical University, Varna, Bulgaria; 2 Department of Nuclear Medicine, Metabolic Therapy and Radiotherapy, Varna Medical University, Varna, Bulgaria

**Keywords:** Prostatic Neoplasms, Positron-Emission Tomography, Lymph Nodes, Diagnosis

*Dear Editor*,

We read with great interest the paper, published in Nephrology-Urology Monthly (1). Prostate cancer (PCa) is the most common solid malignancy of elderly males. Therefore, it should be suspected as the primary malignant lesion in elderly men with spread metastases of unidentified origin, especially when the bones are involved.

The primary landing site of PCa metastases are the internal iliac lymph nodes (LNs) (2). After the pelvic LNs, the lymphatic spread of the tumor cells continues upwards, involving the paracaval and paraaortic LNs, and then the supradiafragmatic LNs. Diffuse lymphatic spread, involving the cervical, supraclavicullar and the axillary LNs, as described in this case, is very rare, and occurs when the disease is too advanced and the tumor - too aggressive.

The primary diagnosis of PCa is based on three simple diagnostic tools: the digital rectal examination (DRE), the prostate-specific antigen (PSA) testing and the transrectal ultrasound. If any of these is abnormal, a prostate biopsy is indicated to confirm the clinical suspicion of PCa. In this case, however, the DRE and PSA test had been used as secondary diagnostic tools, although they could raise the suspicion of PCa much earlier. The extremely high PSA reported (4020 ng/mL) is clearly indicative of metastatic PCa.

FDG PET/CT is a new imaging modality used to detect cancer and monitor the response to therapy. With regard to PCa detection, FDG PET/CT has limited value: firstly, because there is a very low tracer avidity, as only a few (around 1%) PCa lesions are FDG avid, and secondly, because the majority of cases, demonstrating focal FDG uptake in the prostate, are benign (3). Besides, the evaluation of the regional pelvic LN metastases is severely limited because of the high bladder tracer activity.

On the other side, however, there is evidence in the literature that FDG PET/CT sensitivity and positive predictive value to detect PCa raises with Gleason score 7 or greater, reaching up to 80% and 87%, respectively (4). The reported case also confirms these findings. High FDG avidity had been demonstrated in LNs, bones, and the prostate. This is most probably due to the aggressive tumor biology (Gleason score of 9/5+4/).

FDG PET/CT is inferior to bone scintigraphy in detecting osseous metastases, but it plays an important role in identifying soft-tissue metastases, like LN and liver metastases. Differential diagnosis of generalized LN metastases includes various types of tumors, with lymphomas staying on the first place. Although rare, lymphomas of the prostate, are also described, and should be taken into consideration (5).

PCa cells initially respond favorably to androgen deprivation therapy, but sooner or later they become androgen-independent. In this case, FDG PET/CT proved to be an excellent tool to detect both the primary cancer and the metastatic tumor sites, and to monitor the response to hormonal treatment. It would be interesting to follow the case further on, and to assess the usefulness of FDG PET/CT to monitor treatment, not only during the initial, androgen-dependent, but also during the hormone-refractory stage of the disease.
